# Characterization of a New SCC*mec* Element in *Staphylococcus cohnii*

**DOI:** 10.1371/journal.pone.0014016

**Published:** 2010-11-17

**Authors:** Zhiyong Zong, Xiaoju Lü

**Affiliations:** 1 Center of Infectious Diseases, West China Hospital, Sichuan University, Chengdu, China; 2 Division of Infectious Diseases, State Key Laboratory of Biotherapy, Chengdu, China; National Institute of Allergy and Infectious Diseases, United States of America

## Abstract

**Background:**

Many SCC*mec* elements of coagulase-negative staphylococci (CoNS) could not be typed using multiplex PCR. Such a ‘non-typable’ SCC*mec* was encountered in a *Staphylococcus cohnii* isolate.

**Methodology/Principal Findings:**

The SCC*mec* type of methicillin-resistant *S. cohnii* clinical isolate WC28 could not be assigned using multiplex PCR. Newly-designed primers were used to amplify *ccrA* and *ccrB* genes. The whole SCC*mec* was obtained by three overlapping long-range PCR, targeting regions from left-hand inverted repeat (IRL) to *ccrA/B*, from *ccrA/B* to *mecA* and from *mecA* to orfX. The region abutting IRL was identified using inverse PCR with self-ligated enzyme-restricted WC28 fragments as the template. WC28 SCC*mec* had a class A *mec* gene complex (*mecI*-*mecR1*-*mecA*). The *ccrA* and *ccrB* genes were closest (89.7% identity) to *ccrA*_SHP_ of *Staphylococcus haemolyticus* strain H9 and to *ccrB3* (90% identity) of *Staphylococcus pseudintermedius* strain KM241, respectively. Two new genes potentially encoding AAA-type ATPase were found in J1 region and a ψTn*554* transposon was present in J2 region, while J3 region was the same as many SCC*mec* of *Staphylococcus aureus*. WC28 SCC*mec* abutted an incomplete SCC element with a novel allotype of *ccrC*, which was closest (82% identity) to *ccrC1* allele 9 in *Staphylococcus saprophyticus* strain ATCC 15305. Only two direct target repeat sequences, one close to the 3′-end of orfX and the other abutting the left end of WC28 SCC*mec*, could be detected.

**Conclusions/Significance:**

A new 35-kb SCC*mec* was characterized in a *S. cohnii* isolate, carrying a class A *mec* gene complex, new variants of *ccrA5* and *ccrB3* and two novel genes in the J1 region. This element is flanked by 8-bp perfect inverted repeats and is similar to type III SCC*mec* in *S. aureus* and a SCC*mec* in *S. pseudintermedius* but with different J1 and J3 regions. WC28 SCC*mec* was arranged in tandem with an additional SCC element with *ccrC*, SCC_WC28_, but the two elements might have integrated independently rather than constituted a composite. This study adds new evidence of the diversity of SCC*mec* in CoNS and highlights the need for characterizing the ‘non-typable’ SCC*mec* to reveal the gene pool associated with *mecA*.

## Introduction

Coagulase-negative staphylococci (CoNS) are opportunistic pathogens [1] and are usually resistant to methicillin [2]. In staphylococci, methicillin resistance is mainly dependent on the expression of the *mecA* gene, which encodes PBP2a, a transpeptidase with a low affinity for β-lactams [3]–[4]. *mecA* together with its regulatory genes and associated insertion sequences forms the *mec* gene complex, which is carried by a mobile genetic element (MGE) termed the staphylococcal cassette chromosome *mec* (SCC*mec*) [5]. SCC*mec* is bounded by terminal inverted repeats (IRs) and integrates site specifically in the staphylococcal chromosome close to the 3′ end of orfX [6], a gene of unknown function located close to the origin of the chromosomal replication. The integrate site sequence (ISS) usually contains the consensus sequence GA(A/G)GC(A/G/T)TATCA(C/T)AA(A/G)T(A/G)(A/G) [7]–[8]. A 15 bp sequence is duplicated as direct target repeats (DR) on insertion of SCC*mec* [6]–[7]. Integration and excision of SCC*mec* are due to recombinases encoded by a set of cassette chromosome recombinase (*ccr*) genes (*ccrC* or the pair of *ccrA* and *ccrB*) [6], [9]. The *ccr* gene(s) and surrounding genes constitute the *ccr* gene complex [6], [9]. In addition to *ccr* and *mec* gene complexes, SCC*mec* contains a few other genes, many of which have unknown functions, and various other MGE, e.g. insertion sequences, transposons and plasmids. These genes and MGE are located in three joining regions, i.e. J1 between the left-hand IR (IRL) and the *ccr* gene complex, J2 between the *ccr* and *mec* gene complexes, and J3 between the *mec* gene complex and the right-hand IR (IRR) [9].

Eight types (I to VIII) of SCC*mec* have been assigned for *Staphylococcus aureus* based on the classes of the *mec* gene complex and the types of the *ccr* gene complex [9]. As methicillin resistance is more prevalent in CoNS than in *S. aureus*, CoNS may serve as a larger reservoir of SCC*mec* available for *S. aureus* to form methicillin-resistant *S. aureus* (MRSA) [6]. However, compared to MRSA, much less is known about the genetics of *mecA* in CoNS [10]. According to the available data [10]–[21], SCC*mec* elements are more diverse in CoNS, with new variants of *ccr* genes continuing to be identified [13], [20]–[22]. Although type III and IV SCC*mec* are prevalent in CoNS, many SCC*mec* elements of CoNS could not be typed using currently-available schemes based on multiplex PCR [6], [21]. In a study of SCC*mec* in local CoNS clinical isolates, a *Staphylococcus cohnii* isolate containing a “non-typeable” SCC*mec* was encountered. This “non-typeable” SCC*mec* was characterized in detail and is reported here.

## Methods

### Strain and SCC*mec* typing

CoNS isolate WC28 was recovered from a clinical specimen (wound secretion) collected in West China Hospital, Chengdu, western China. This isolate was identified as *S. cohnii* by partially sequencing the 16s rRNA gene amplified with the universal primers 27F and 1492-R (Table 1) [23]. WG28 could grow on plates containing 4 µg/ml cefoxitin (Sigma, St Louis, MO). The *mecA* gene and its regulatory genes *mecI* and *mecR1* were detected by PCR as described previously [24]. The SCC*mec* typing was carried out using multiplex PCR as described previously [24].

**Table 1:** Primers used for PCR.

Primer	Sequence (5′-3′)^a^	Target/location^b^	Reference
27F	GGTTACCTTGTTACGACTT	16s rRNA gene	[23]
1492R	AGAGTTTGATCCTGGCTCAG		[23]
MecA147-F	GTGAAGATATACCAAGTGATT	*mecA*	[24]
MecA147-R	ATGCGCTATAGATTGAAAGGA		[24]
mecI-F	CCCTTTTTATACAATCTCGTT	*mecI*	[24]
mecI-R	ATATCATCTGCAGAATGGG		[24]
ccrA-UF1	AATGTGAHGTATTATGTTGYTA	*ccrA*	This study
ccrA-UR1	GGTTCATTTTTDAARTAGAT		This study
ccrB-UF1	CGTGTATCAACDGAAATVCAA	*ccrB*	This study
ccrB-UR1	CTTTATCACTTTTGAYWATTTC		This study
orfX-F1	GAAAAAGCACCWGAAAMTATGAG	orfX	This study
IRL-scc	TATCRGWTRATGATGMGGTTT	IRL of SCC*mec*	This study
ccrA_28-R1	TGATTGATGACACGACCACA	*ccrA*	This study
28-7	TTCCTCCTTCATTCCTCTGG	orf2	This study
Tn554-UR1	TTCTATGGCAGAAGGATGTGG	ψTn*554*	This study
28-10	AATTGGATGTCAACGTACAGG	5′ end of orf15	This study
HMG-up	ATTGTGCTTGATGAGCTTGG	3′ end of orf19	This study
28-11	CCATCTGTGGAGCCTTTTGT	orfA	This study
orf28-F1	TTGCCAATTAAAAGGTTGGTT	orfL	This study
orf28-R1	GCACAACCCCGTAACCTACT	orfL	This study
orf28-R2	ATTTTCACCACGCTCCATTT	orfL	This study
28-14	GCAGGTGTTATTGGACACGA	orfB	This study
28-17	TTTCGTTTCTCACTACCATTTG	orfC	This study
28-18	TGGTAGGTCCTTTCGTAGAAGA	orfC	This study
28-21	CGTACAAAATAAGCCCACGA	orfF	This study
28-22	CCATGCAGATCGAAAAGGTA	orfF	This study
28-23	CCGAAATCTGTAGTGCGTCA	*ccrC*-orfF spacer	This study
28-24	GGAACAATCAGAGCGTGGA	*ccrC*	This study
28-13	TTGAGCATCTCCGTTTCTTTC	orf3	This study
28-32	ACACCAATCAACCTCAAGCA	orfI	This study
28-26	ACGTTTCACAGCCCAATTTT	*ccrC*	This study
28-39	CCAAGCGATCAACAGACAAC	upstream of orfN	This study

a D: A, G or T; H: A, C or T: M: A or C; R: A or G; W: A or T; Y: C or T; V: A, C or G.

b Description of orfs are available in Table S1 and 3.

### Identification of *ccr* genes

Since primers targeting *ccrAB1*, *ccrAB2*, *ccrAB3* and *ccrC* [24] failed to detect the *ccr* genes in WC28. *ccrA* and *ccrB* of WC28 were obtained using new primers (Table 1) designed from an alignment of known *ccrA* and *ccrB* sequences retrieved from GenBank.

### PCR mapping

**Figure 1:**
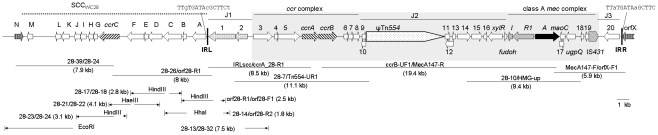
Structure of and PCR mapping for WC28 SCC*mec* and adjacent regions. Numbers and alphabets represent gene names in SCC*mec* (listed in Table S1) and SCC_WC28_ (listed in Table 3), respectively. ψTn*554* contains *tnpB*, *tnpC*, *cadC* and *cadB*. The 15 bp sequences abutting the IR are shown with nucleotides that differ in lower case. The region similar to type III SCC*mec* (85/2082) and the SCC*mec* of *S. pseudintermedius* KM241 is highlighted with a grey background. PCR primers and amplicon sizes are indicated. Several self-ligated restricted fragments were used as templates for inverse PCR with the names and restriction locations of the enzymes being shown.

Three overlapping long-range PCR (Fermentas, Burlington, ON, Canada; Figure 1) were used to obtain the whole SCC*mec* and to confirm the links between different genetic components. These three PCR linked IRL to *ccrA*, the *ccrAB* genes to *mecA*, and *mecA* to orfX (Figure 1).

### Inverse PCR

A few inverse PCR reactions were employed to identify the region abutting IRL with pairs of outwards-facing primers (Table 1 and Figure 1). Genomic DNA of WC28 prepared using a commercial kit (Tiangen, Beijing, China) was restricted with a restriction enzyme (Figure 1), self-ligated with T4 DNA ligase (New England Biolabs, Ipswich, NY, USA) and then used as a template for inverse PCR. The links between genetic elements were confirmed by overlapping long-range PCR (Figure 1, primers listed Table 1).

### Sequencing

Amplicons were sequenced by primer walking using an ABI 3730xl DNA Analyzer (Applied Biosystems, Foster City, CA) at the Beijing Genomics Institute (Beijing, China). Sequences were assembled using the SeqMan II program in the Lasergene package (DNASTAR Inc, Madison, WI) and similarity searches were carried out using BLAST programs (http://www.ncbi.nlm.nih.gov/BLAST/).

#### Nucleotide sequences accession number

The complete sequence of the WC28 SCC*mec* is deposited in GenBank as GU370073.

## Results and Discussion

WC28 contained *mecA* gene but its SCC*mec* type could not be assigned using multiplex PCR, suggesting that WC28 might harbor a new SCC*mec* element.

### WC28 SCC*mec* had perfect IRs but imperfectly-matched abutting sequences

IRs vary in size and can be imperfect in different SCC*mec* [6]–[7]. Nonetheless, the IRs of SCC*mec* type I (strain NCTC10442), II (N315), III (85/2082) and IVa (CA05) in *S. aureus* contain a consensus 8-bp sequence GC(A/G/T)TATCA at the end [7], [25]. In WC28 GCTTATCA bounded the SCC*mec* and constituted the 8-bp perfect IR. The 15-bp sequences abutting both ends of the WC28 SCC*mec* were not perfectly matched, with three nucleotide differences (Figure 1), suggesting that the WC28 SCC*mec* might have been formed by recombination. However, based on SCC*mec* excision experiments [7], it appears that nucleotide mutations are likely to be introduced during the insertion of SCC*mec*, generating target repeats that are not perfectly matched. The 15-bp sequences abutting the WC28 SCC*mec* may therefore be slightly different simply as a result of direct insertion of this element in orfX.

### WC28 SCC*mec* carried a class A *mec* gene complex

The SCC*mec* of WC28 had a class A *mec* gene complex composed of *mecA*, *mecI mecR1*, several other genes and a single copy of insertion sequence IS*431* downstream of *mecA* (Figure 1 and Table S1 in Online Supporting Information). The class A *mec* gene complex is also present in SCC*mec* types II, III and VIII and SCC*mec* of unassigned types in *Staphylococcus pseudintermedius* strain KM241 [21] and *Staphylococcus saprophyticus* strain TSU33 [20]. The class A *mec* gene complex in WC28 was most similar to that in *S. saprophyticus* TSU33 with only two nucleotide differences.

### New variants of *ccrA* and *ccrB* representing challenges for the present classification scheme

The WC28 SCC*mec* contained a *ccr* gene complex with new *ccrA* and *ccrB* variants. The WC28 *ccrB* gene (*ccrB*_WC28_) was 1503 bp in length, shorter than most other *ccrB* genes (1629 bp) reported previously [9]. *ccrB*_WC28_ was most similar (90% identity) to *ccrB3* (*S. pseudintermedius* KM241) [21] and was 88.9% identical to *ccrB*_SHP_ (*Staphylococcus haemolyticus* H9) [13] and 88.7% to *ccrB3* (*S. aureus* 85/2082) [7] (Table 2). According to the guidelines for reporting novel SCC*mec* elements [9], *ccr* genes with greater than 85% nucleotide identity should be classified into the same allotype. *ccrB*_WC28_ is therefore a new variant of *ccrB3*.

**Table 2:** Comparison of *ccrA*_WC28_, *ccrB*_WC28_ and *ccrC*_WC28_ with selected *ccr* genes.

*ccr* allotype	Species & strain	Accession no.	% identity		
			*ccrA*_WC28_	*ccrB*_WC28_	*ccrC*_WC28_
*ccrA5* a	*S. haemolyticus* H9	EU934095	89.7		
*ccrA3*	*S. aureus* 85/2082	AB037671	85.7		
*ccrA5*	*S. pseudintermedius* KM241	AM904731	85.0		
*ccrA1*	*S. aureus* NCTC10442	AB033763	77.1		
*ccrA2*	*S. aureus* N315	D86934	74.2		
*ccrA4*	*S. aureus* HDE288	AF411935	64.0		
*ccrB3* b	*S. pseudintermedius* KM241	AM904731		90.0	
*ccrB3* c	*S. haemolyticus* H9	EU934095		88.9	
*ccrB3*	*S. aureus* 85/2082	AB037671		88.7	
*ccrB6*	*S. saprophyticus* ATCC15305	NC_007350		82.9	
*ccrB2*	*S. aureus* N315	D86934		81.0	
*ccrB7*	*S. saprophyticus* STU33	AB353724		81.0	
*ccrB1*	*S. aureus* NCTC10442	AB033763		76.9	
*ccrB4*	*S. aureus* HDE288	AF411935		72.7	
*ccrC1* allele 9	*S. saprophyticus* ATCC15305	NC_007350			82.3
*ccrC1* allele 1	*S. aureus* JCSC3624(WIS)	AB121219			81.3
*ccrC1* allele 4	*S. aureus* M	U10927			80.8
*ccrC1* allele 6	*S. haemolyticus* 25–60	EF190467			80.6
*ccrC1* allele 5	*S. aureus* JCSC1435	AP006716			80.4
*ccrC1* allele 10	*S. aureus* UMCG-M4	GQ902038			80.2
*ccrC1* allele 8	*S. aureus* PM1	AB462393			80.1
*ccrC1* allele 2	*S. aureus* TSGH17	AY894416			80.1
*ccrC1* allele 7	*S. epidermidis* 13–48	EF190468			79.9
*ccrC1* allele 3	*S. aureus* 85/2082	AB037671			79.9

a Originally reported as *ccrA*_SHP_, 86.6% identical to *ccrA5* (KM241).

b Originally reported as *ccrB5* but re-designated *ccrB3* [9], 91.4% identical to *ccrB3* (85/2082).

c Originally reported as *ccrB*_SHP_, 87.1% identical to *ccrB3* (KM241) and 85.9% to *ccrB3* (85/2082).

The WC28 *ccrA* gene (*ccrA*_WC28_; 1350 bp) had the highest identity (89.7%) with *ccrA*_SHP_ (*S. haemolyticus* H9) and was 85.7% identical to *ccrA3* (85/2082) and 85.0% to *ccrA5* (*S. pseudintermedius* KM241) (Table 2). It appears that *ccrA*_WC28_ could be a member of the *ccrA3* or *ccrA5* allotype, illustrating a problem with the current classification system [9]. Nonetheless, *ccrA*_SHP_, the closest match to *ccrA*_WC28_, is closer to *ccrA5* (KM241) than to *ccrA3* (85/2082; 86.6 vs 80.3% identity), and therefore should be clustered with *ccrA5* based on the 85% cutoff value. Accordingly, it seems more appropriate that *ccrA*_WC28_ should be designated as the *ccrA5*, rather than the *ccrA3,* allotype. Like *S. haemolyticus* H9 and *S. pseudintermedius* KM241, WC28 had a *ccrA5B3* type *ccr* gene complex, different from all *ccr* complex types identified in *S. aureus* so far.

Compared with those in *S. aureus*, the *ccrAB* sequences in CoNS appear to be more diverse with several new variants reported recently [6], [13], [20]–[21]. *ccrAB* sequences in CoNS could have more than 85% identity with more than one designated allotype, exemplified by *ccrA*_WC28_ here and *ccrB3* of *S. pseudintermedius* KM241, which is 91.4% identical to *ccrB3* (85/2082) and 85.5% to *ccrB1* (*S. aureus* MSSA476). This dilemma may need to be considered when developing the classification guidelines for SCC*mec* in CoNS. It seems reasonable to assign a *ccr* variant to its closest allotype when it had more than 85% identity with two or more designated allotypes.

### The joining regions in WC28 SCC*mec* contained several new features

Five genes were identified between IRL of SCC*mec* and *ccrA*. The three genes adjacent to *ccrA* were similar to the counterparts in *S. pseudintermedius* KM241 and appear to be part of the *ccr* gene complex. The remaining two genes (orf1 and −2) closest to IRL had no significant matches with any staphylococcal sequences currently deposited in GenBank but had the highest identities to a gene (lwe0773; 62% identical to orf1) in *Listeria welshimeri* SLCC5334 (NC_008555) and a gene (MSC_1061; 64% identical to orf2) in *Mycoplasma mycoides* PG1 (NC_005364). These two genes are likely to encode proteins of the AAA-type ATPase superfamily. AAA refers to ATPases associated diverse cellular activities such as protein degradation and intercellular transport [26]. The presence of these two novel genes suggests that the J1 region in the WC28 SCC*mec* is different from those reported previously.

Like SCC*mec* type III of *S. aureus* 85/2082 and the SCC*mec* of *S. pseudintermedius* KM241, the *ccr* and the *mec* gene complexes in the WC28 SCC*mec* were separated by a few genes, most of which have unknown functions, and ψTn*554* carrying cadmium resistance determinants (Table S1 and Figure 1). Of note, there is a single nucleotide deletion in the transposase B gene, *tnpB*, of ψTn*554* in WC28 compared with those reported before. This deletion is not due to an error as it was confirmed by sequencing at both directions. Due to the deletion, two smaller open reading frames instead of a complete *tnpB* gene were present in WC28 but the impact of this deletion on the function of ψTn*554* remains unexplored. In general, this J2 region in the WC28 SCC*mec* is almost identical to those in the KM241 SCC*mec* and SCC*mec* type III (85/2082), except a few nucleotide differences, most of which were in ψTn*554*.

Downstream of the *mec* gene complex, the J3 region of WC28 contained one gene of unknown function (Table S1). The same J3 region has also been seen in many SCC*mec* elements of different types or subtypes, e.g. type I, IIb, IVa and VI in *S. aureus* [9] and an unassigned type in *S. saprophyticus* TSU33 [20]. This structure was termed the downstream constant segment (*dcs*) [9], [27]. Of note, the *dcs* is not present in *S. pseudintermedius* KM241, suggesting that the WC28 and KM241 SCC*mec* had different J3 regions.

### WC28 SCC*mec* abuts another SCC carrying a novel allotype of *ccrC*

A 16 kb region was identified abutting the IRL of WC28 SCC*mec* on one side and abutting a gene, designated orfN here, which putatively specified an FMN-binding flavin reductase on the other side. Variants of this flavin reductase-encoding gene were present in all *S. aureus* and *Staphylococcus epidermidis* genomes available in GenBank, suggesting that this gene was part of the staphylococcal core genome.

A *ccrC* gene was identified in this 16 kb region. All *ccrC* genes identified previously shared more than 87% identity and therefore were variants of a common *ccrC* allotype based on the 85% cutoff value [9]. These variants included *ccrC1* allele 1 (in SCC*mec* V) (Accession no. AB121219), 2 (AY894416), 3 (AB037671) (in SCC*Hg* carrying the mercury resistance operon, adjacent to SCC*mec* III), 4 (U10927), 5 (AP006716), 6 (EF190467), 7 (EF190468), 8 (AB462393), 9 (NC_007350) and 10 (GQ902038) from *S. aureus* and several unassigned *ccrC1* alleles in coagulase-negative staphylococci. The 1677-bp *ccrC* in WC28 was a novel *ccrC* allotype, closest (82% identity) to *ccrC1* allele 9 in *S. saprophyticus* ATCC 15305 and 81% identical to *ccrC1* allele 1 in *S. aureus* (Table 2). Based on the 85% cutoff value [9], *ccrC* in WC28 could be therefore designated *ccrC2* allele 1.

The presence of *ccrC* suggested that this 16 kb region was likely to be a SCC element, therefore designated SCC_WC28_ here, which was arranged in tandem with WC28 SCC*mec*. The presence of two SCC elements in tandem could result from separate integration of the two elements, but the two SCC elements could also constitute a composite generated by fusion of the two elements following deletion of the original junction region containing the DR [9]. Nonetheless, only two DR sequences, one close to the 3′-end of orfX and the other abutting the IRL of WC28 SCC*mec*, could be detected. This suggested that WC28 SCC*mec* and SCC_WC28_ might have integrated independently rather than constituted a composite.

In addition to *ccrC*, SCC_WC28_ contained a few other genes (Table 3), most of which have counterparts seen in SCC*Hg* or in SCC*mec* type V, but function of most of these genes remained undetermined. No MGE such as IS*431* and Tn*4001* were present in SCC_WC28_. Of note, no DR sequences could be detected flanking SCC_WC28_, suggesting that SCC_WC28_ was probably incomplete and the original junction sequence between this element and the core chromosome could have been deleted due to unknown process.

**Table 3:** Genes in SCC_WC28_.

Gene	Position^a^	Product	Closest match
orfA	16947-15829	Hypothetical protein	67% identical to a gene (BCQ_477, function unknown) in *Bacillus cereus* Q1 (CP000227)
orfB	15359-15057	Hypothetical protein	No significant matches
orfC	15046-13550	Hypothetical protein	88% identical to a gene (SSP0042, function unknown) of SCC*mec* in *S. saprophyticus* ATCC15305 (NC_007350)
orfD	13324-12224	Putative DNA/RNA polymorease	79% identical to a gene (function unknown) of SCC*mec* type V, e.g. in *S. aureus* PM1 (ORF no. 25, AB462393)
orfE	12231-11860	Hypothetical protein	94% identical to a gene (function unknown) of SCC*mec* type V, e.g. in *S. aureus* PM1 (ORF no. 26)
orfF	11860-10565	Putative phage/plasmid primase	85% identical to a gene (function unknown) of type V SCC*mec*, e.g. in *S. aureus* PM1 (ORF no. 27)
*ccrC*	9998-8322	CcrC Recombinase	82% identical to *ccrC1* allele 9 in *S. saprophyticus* ATCC15305 (NC_007350)
orfG	8217-7476	Hypothetical protein, DUF 950 superfamily	81% identical to a gene (SSP0034, function unknown) of SCC*mec* in *S. saprophyticus* ATCC15305
orfH	7865-7469	Hypothetical protein, DUF 960 superfamily	84% identical to a gene (function unknown) in SCC*Hg*, e.g. in TW20 (SATW20_00450, FN433596), and also in SCC*mec* in *S. pseudintermedius* KM241 (AM904731) and KM1381 (AM904732)
orfI	7453-6947	Hypothetical protein, DUF 1643 superfamily	84% identical to a gene (function unknown) of SCC*mec* type V, e.g. in *S. aureus* PM1 (ORF no. 11)
orfJ	6965-6477	Putative DNA repair protein, RadC	82% identical to a gene encoding a putative RadC of SCC*mec* in *S. saprophyticus* TSU33 (AB353724)
orfK	6070-5432	Hypothetical protein	79% identical to a gene (SATW20_00450, function unknown) of SCC*Hg* in *S. aureus* TW20
orfL	5394-4927	Hypothetical protein	No significant matches
orfM	3678-3208	Hypothetical protein	84% identical to a gene (SATW20_00490, function unknown) of SCC*Hg* in *S. aureus* TW20

a Positions are according to GenBank accession no. GU370073.

In summary, *mecA* is carried by a 35-kb SCC*mec* in WC28, which has the class A *mec* gene complex and a *ccrA5B3*-type *ccr* gene complex, contains a ψTn*554* and a copy of IS*431* but no plasmids, is flanked by 8-bp perfect IRs and appears to have generated 15-bp DR with nucleotide mutations on insertion. This element in WC28 is a new SCC*mec* since it contains a new *ccr* gene complex and also carries two novel genes in the J1 region. WC28 SCC*mec* was arranged in tandem with an additional SCC element, SCC_WC28_, with a novel *ccrC* allotype, *ccrC2*. However, the two elements might have integrated independently rather than constituted a composite.

**Figure 2:**
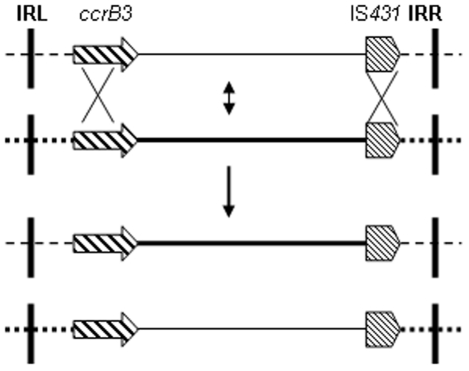
A proposed model for double crossover-mediated exchange between two SCC*mec*. When two different SCC*mec* (not to scale) contain two sequences of homology, exemplified by *ccrB3* and IS*431* here, two homologous recombination events (the upper panel) occurring between the two sequences can result in exchange of the intervening components (lines of different thicknesses) between the two SCC*mec* (the lower panel).

As a whole, the WC28 SCC*mec* is very similar to that of *S. pseudintermedius* KM241 except at both ends (Figure). Based on characteristics of the *mec* and *ccr* gene complexes, the WC28 and KM241 SCC*mec* should be considered together as a new type, while the different J1 and J3 regions suggest that these two SCC*mec* are of two distinct subtypes. The WC28, KM241 and type III (*S. aureus* 85/2082) SCC*mec* share a similar “core” including the *ccr* and *mec* gene complexes and the J2 region suggesting a possible common origin. The divergent J1 and J3 regions in these three SCC*mec* might have resulted from two recombination events occurring in two regions of homology, one of which appears to be IS*431* downstream of *mecA* and another might be *ccrB3* or adjacent sequences (a proposed scheme is shown in Figure 2). The similarity and divergence between SCC*mec* in CoNS and those in *S. aureus* highlights the need to characterize SCC*mec* elements in CoNS, particularly those not identified by PCR-based typing schemes. The information generated is essential for revealing the potential reservoir of components that could allow formation of diverse elements carrying *mecA* and for appreciating the origin and the evolution of SCC*mec*.

## Supporting Information

Table S1 Genes in the WC28 SCC*mec*.
(0.10 MB DOC)
