# Supplementary material for: Characterization of a New SCCmec Element in Staphylococcus cohnii
Source: PLoS One. 2010 Nov 17;5(11):e14016. doi: 10.1371/journal.pone.0014016 (PMC2984492; doi:10.1371/journal.pone.0014016)
Supplement: Table S1 — Genes in the WC28 SCCmec. (0.10 MB DOC) [file pone.0014016.s001.doc]

**Table S1.** Genes in the WC28 SCC*mec*

| Name | Position*^a^* | Product | Homology to selected SCC*mec* types*^b,c^* | | |
| --- | --- | --- | --- | --- | --- |
|  |  |  | (KM241) | (TSU33) | III (85/2082) |
| orf1*^d^* | 19379-17166 | Putative AAA-ATPase | - | - | - |
| orf2*^e^* | 20421-19372 | Putative AAA-ATPase | - | - | - |
| orf3 | 22444-20894 | Hypothetical protein | orf241-1 (86) | - | - |
| orf4 | 22625-22915 | Hypothetical protein | orf241-2 (94) | NS | NS |
| orf5 | 22915-24708 | Putative membrane protein containing Superfamily II helicase domain | orf241-3 (89) | NS | NS |
| *ccrA* | 24887-26236 | CcrA recombinase | *ccrA5* (85) | *ccrA* (77) | *ccrA3* (85) |
| *ccrB* | 26258-27760 | CcrB recombinase | *ccrB5* (90) | *ccrB* (81) | *ccrB3* (88) |
| orf6 | 28357-28707 | Hypothetical protein | orf241-6  (100) | 14588-14250 (80) | Z011  (100) |
| orf7 | 28708-29103 | Hypothetical protein | orf241-7 & -8 (100)*^f^* | 14154-13843 (77) | Z013  (100) |
| orf8 | 29122-29643 | Hypothetical protein | orf241-9 (100) | NS | Z014 (100) |
| orf9 | 30161-29867 | Hypothetical protein | NA (100) | NS | CZ008 (100) |
| orf10 | 29845-30168 | DNA repair protein RadC | orf241-11 (100) | NS | NA (100) |
| *tnpB*∆1*^g^* | 30363-31496 | ψTn*554* transposase B | *tnpB* (99) | - | *tnpB* (99) |
| *tnpB*∆2*^g^* | 31535-32254 | ψTn*554* transposase B | *tnpB* (99) | - | *tnpB* (99) |
| *tnpC* | 32261-32638 | ψTn*554* transposase C | *tnpC* (98) | - | *tnpC* (98) |
| *cadC* | 32876-33241 | Cadmium resistance  protein C | *cadC* (99) | - | *cadC* (99) |
| *cadB* | 33234-35648 | Cadmium resistance  protein B | *cadB* (99) | - | *cadB* (99) |
| DS RF | 35687-36346 | Cadmium resistance transporter | DS RF (100) | - | DS RF (100) |
| orf11 | 36564-37085 | Hypothetical protein | NA (100) | - | Z023 (100) |
| orf12 | 37258-36614 | Putative membrane protein | orf24-12 (100) | - | CZ021 (100) |
| orf13 | 37524-37258 | Hypothetical protein containing regulator protein FrmR domain | orf24-13 (100) | - | CZ022 (100) |
| orf14 | 37654-38718 | Putative NADH dehydrogenase | orf24-14 (100) | - | Z024 (100) |
| orf15 | 38830-39678 | Metallo-β-lactamase  family protein | orf24-15 (99) | - | Z025 (99) |
| orf16 | 39825-40085 | Metallo-β-lactamase  family protein | orf24-16 (100) | - | Z026 (100) |
| *xylR* | 41347-40199 | Xylose repressor | *xylR* (100) | - | *xylR* (100) |
| *fudoh* | 41719-41507 | A protein suppressing colony spreading and virulence expression | NA (99) | NA (99) | CZ026 (99) |
| *mecI* | 42191-41820 | Methicillin resistance regulatory protein | *mecI*  (100) | *mecI*  (100) | *mecI*  (99) |
| *mecR1* | 43948-42191 | Signal transducer protein | *mecR1*  (99) | *mecR1*  (100) | *mecR1^h^*  (99) |
| *mecA* | 44048-46054 | Penicillin binding protein 2a | *mecA* (100) | *mecA* (100) | *mecA* (99) |
| orf17 | 46047-46430 | Hypothetical protein | NA (100) | NA (100) | Z031 (100) |
| *maoC* | 46528-46100 | Putative acyl dehydratase MaoC | orf24-17  (100) | 4987-5415 (99) | CZ029  (100) |
| *ugpQ* | 47368-46625 | Glycerophosphoryl diester phosphodiesterase | *ugpQ*  (100) | 4148-489*1* (99) | *ugpQ*  (100) |
| orf18 | 48067-47825 | Putative membrane protein | NA (99) | NA (100) | NA (98) |
| orf19 | 48554-48165 | Putative HMG-CoA synthase (partial) | orf24-18  (100) | 2962-3351 (100) | CZ032  (100) |
| *tnpA* | 48590-49264 | IS*431* Transposase | *tnpA* (99) | *tnpA* (100) | *tnpA* (100) |
| orf20 | 51084-49789 | Hypothetical protein | - | 432-1727 (100) | - |
| orfX (partial) | 51384-513  67 | Hypothetical protein | NS | NS | orfX (99) |

*^a^* Positions are according to GenBank accession no. GU370073.

*^b^* Strain nos. are indicated in brackets. The SCC*mec* types for *S. pseudintermedius* KM241 (AM904731) and *S. saprophyticus* TSU33 (AB353724) are not assigned.

*^c^* Gene names are listed with nucleotide identity (100%) indicated in brackets. In most cases, no gene names were given in the GenBank entry of TSU33, so the positions are shown instead. NS, no significant matches (similar sequences are present but display less than 75% identity).-, not present. NA, not annotated in the corresponding GenBank entries.

*^d^* 62% identical to lwe0773 (*Listeria welshimeri* SLCC5334; NC_008555).

*^e^* 64% identical to MSC_1061 (*Mycoplasma mycoides* PG1; NC_005364).

*^f^* orf7 is annotated as two orfs in KM241.

*^g^* Compared with those reported before, there is one nucleotide, G, deletion between locations 15920 and 15921 in *tnpB* of WC28, resulting in two *tnpB* fragments, ∆1 and ∆2.

*^h^* There is a 166-bp deletion in *mecR1* of 82/2082.
